# Olfactory information processing viewed through mitral and tufted cell-specific channels

**DOI:** 10.3389/fncir.2024.1382626

**Published:** 2024-03-08

**Authors:** Tatsumi Hirata

**Affiliations:** Brain Function Laboratory, National Institute of Genetics, SOKENDAI, Mishima, Japan

**Keywords:** olfactory system, tufted cell, mitral cell, mouse, neurogenic tagging, parallel processing

## Abstract

Parallel processing is a fundamental strategy of sensory coding. Through this processing, unique and distinct features of sensations are computed and projected to the central targets. This review proposes that mitral and tufted cells, which are the second-order projection neurons in the olfactory bulb, contribute to parallel processing within the olfactory system. Based on anatomical and functional evidence, I discuss potential features that could be conveyed through the unique channel formed by these neurons.

## Introduction

From a neurodevelopmental perspective, the timing of neuronal birth determines their permanent phenotypes (Hirata and Iwai, 2019), including morphology, physiology and connection patterns (Leone et al., 2008; Fame et al., 2011; Suzuki and Hirata, 2013). Thus, this neurodevelopmental principle should form the functional basis of the brain. We hypothesized that if projection neurons of the olfactory bulb are classified neurodevelopmentally, we might be able to find a wiring logic of olfactory circuits. Chronologically ordered arrangement of olfactory bulb axons in the lateral olfactory tract (Inaki et al., 2004; Yamatani et al., 2004) further encouraged us to take this approach, even though the link between the chronotopic arrangement of axon shafts and the final destinations of their collateral branches remained unclear. These provided the springboard for our dissection of olfactory circuits using neurogenic tagging. Based on our and others’ findings, I will discuss the potential logic of olfactory information processing.

## Logic of olfactory information processing

The anatomical principle of the peripheral olfactory system is feature detection of odorant molecules; olfactory sensory neurons that express the same odorant receptor converge their axons onto a few fixed glomeruli of the olfactory bulb, thereby constructing the stereotypical odor map (Mori et al., 2006; Mori and Sakano, 2011). This odor map is then transferred to the next targets by two major projection neurons, mitral cell (MC) and tufted cell (TC) in the main olfactory bulb (Mori and Sakano, 2021). Their projections are often described as diffuse and widespread (Ghosh et al., 2011; Miyamichi et al., 2011; Sosulski et al., 2011). While specific odorant information sometimes appears over-represented in a few target areas (Miyamichi et al., 2011; Inokuchi et al., 2017), the spatial odor map across the olfactory bulb is basically lost in most of the olfactory target areas due to non-topographic projections.

This strategy seems somewhat exceptional as a sensory system. Although external information is typically represented as a spatial map in many sensory systems, the maps are usually transferred sequentially to higher centers by the labeled-line principle (Kaas, 1997; Cang and Feldheim, 2013). By contrast, the odor map degrades rapidly. This has led to the assumption that olfactory information processing relies on indiscriminate integration of odorant information by mixing projections from the peripheral odor map (Davison and Ehlers, 2011).

This review argues that MCs and TCs offer an alternative perspective: parallel processing in the olfactory system. The parallel processing is another common strategy of the sensory system (Young, 1998). As exemplified by the visual system, different features of information are extracted from the original map and sent to separate target areas in parallel (Nassi and Callaway, 2009), thereby sharpening and enhancing specific features for increased biological significance. While olfactory information features remain elusive, I propose to discuss potential features that MC and TC channels can convey in the olfactory system based on previous observations (Mori and Sakano, 2021).

## MCs and TCs in the olfactory system

Around 20 MCs and 50 TCs relay information received by each glomerulus to higher brain centers (Nagayama et al., 2014). These two projection neuron types occupy distinct layers of the main olfactory bulb and exhibit morphological differences (Mori et al., 1983; Orona et al., 1984). Furthermore, they fire action potentials at different phases of the respiratory cycle (Fukunaga et al., 2012; Igarashi et al., 2012). Electrophysiological analyses suggested that MCs are highly tuned for detection of specific odorants, whereas TCs respond more broadly to a wider range of stimuli (Schneider and Scott, 1983; Ezeh et al., 1993; Nagayama et al., 2004; Griff et al., 2008). Thus, MCs and TCs seem well-poised to convey different kinds of information extracted from the same glomeruli.

Although MCs and TCs exhibit molecular differences (Tepe et al., 2018; Zeppilli et al., 2021), clear discrimination based on gene expression has proven elusive. Previous studies indicated that MCs are born earlier than TCs (Hinds, 1968; Bayer, 1983; Grafe, 1983). I conceived that the birthdate difference can be used to effectively separate these populations. While a study demonstrated differential labeling of olfactory bulb neurons based on birth timing by *in utero* electroporation, this technique only revealed heterogeneous MC populations (Imamura et al., 2011; Imamura and Greer, 2015; Chon et al., 2020). Therefore, we opted for a different genetic method, neurogenic tagging, which allows for separate visualization and manipulation of MCs and TCs based on their distinct birthdates (Hirata et al., 2019).

## Neurogenic tagging of olfactory projection neurons

**Figure 1:**
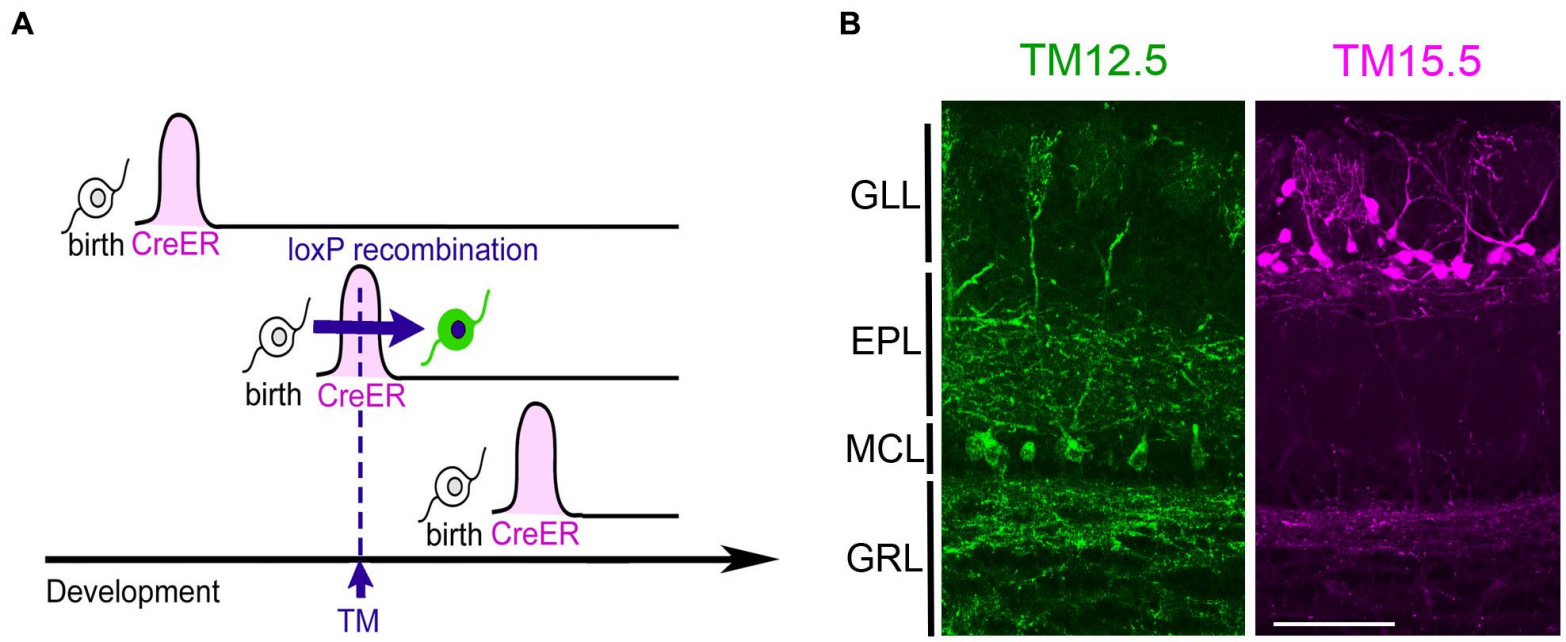
Neurogenic tagging of MCs and TCs. **(A)** A diagram illustrating neurogenic tagging. In the driver mice, tamoxifen (TM)-inducible CreER is transiently expressed during a short time window soon after neuronal birth. A single injection of TM during the neurodevelopmental stage induces *loxP* recombination only in the cells expressing CreER. Modified from Hirata et al. (2021). **(B)** MCs labeled with green fluorescent protein (green, left) and TCs labeled with tdTomato (magenta, right) in the mouse olfactory bulb at postnatal day 21. TM was injected at embryonic day 12.5 (TM12.5) and 15.5 (TM15.5). The complete genotypes of the mice are Neurog2^CreER^(G2A); Cdhr1^tTA^; ROSA26-TRE^mGFP^ for TM12.5 and Neurog2^CreER^(G2A); Cdhr1^tTA^; TRE^tdTomato-sypGFP^ for TM15.5 [see Hirata et al., 2019 for details]. Bar = 100 μm. GLL, glomerular layer; EPL, external plexiform layer; MCL, mitral cell layer; GRL, granule cell layer.

The neurogenic tagging method uses a driver mouse line in which tamoxifen (TM)-inducible Cre recombinase, CreER is expressed only transiently for a short time window immediately after neuronal fates are committed (Figure 1). For this purpose, CreER is driven under the enhancer/ promoter of neural differentiation genes such as neurogenins and neuroDs using the bacterial artificial chromosome transgenic approach (Hirata et al., 2021). A single TM dose at a specific developmental stage induces loxP recombination only in the cells soon after neuronal commitment. These “tagged” neurons are then susceptible to various experimental manipulations using recombination-dependent reporters or effectors. Several driver lines are available for neurogenic tagging (Hirata et al., 2021). Representative images of tagged neurons across the brain by all the drivers are open in public in the NeuroGT database,^1^ visualized using a global neuron-specific reporters (Tau^mGFP-nLacZ^, Hippenmeyer et al., 2005, Jax#021162).

**Figure 2:**
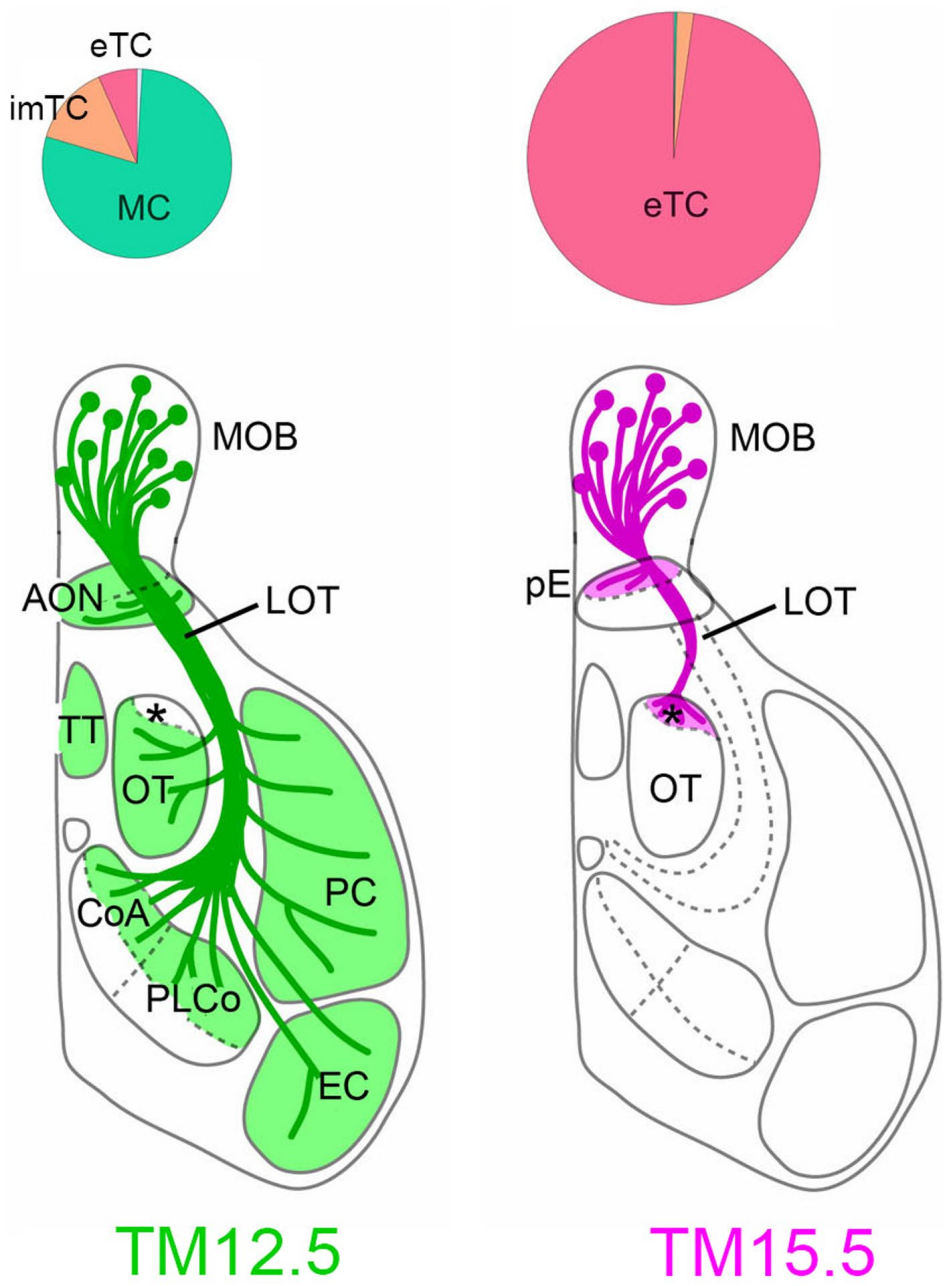
Projections of MCs and TCs revealed by neurogenic tagging. Pie charts on the top show the proportion of neuron types neurogenic-tagged. MCs and TCs are preferentially labeled when TM was injected at embryonic day 12.5 (TM12.5. left) and 15.5 (TM15.5, right), respectively. The diameter of the charts reflects the number of neurons. The brain diagrams on the bottom summarize axon projections of TM2.5 and TM15.5-tagged neurons. The asterisk indicates aiCAP within the olfactory tubercle. imTC, internal and middle TC; eTC, external TC; MOB, main olfactory bulb; AON, anterior olfactory nucleus; pE, pars externa; LOT, lateral olfactory tract; TT, tenia tecta; OT, olfactory tubercle; CoA, cortical amygdala; PLCo, posterolateral cortical amygdala; PC, piriform cortex; EC, entorhinal cortex. Modified from Hirata et al. (2021).

Among the driver lines, the Neurog2^creER^ (G2A) driver is ideal for studying olfactory projection neurons (Hirata et al., 2019). While this method labels a mixture of multiple projection neuron types, MCs and TCs are the major populations when tamoxifen is injected at E12.5 and E15.5, respectively (Figure 2). TCs are typically categorized further as internal, middle, and external subtypes based on their location within the olfactory bulb layer (Mori et al., 1983; Orona et al., 1984). However, in the analysis using neurogenic tagging mice, external TCs far outnumber the other TC subtypes (Hirata et al., 2019). Therefore, this review will primarily focus on external TCs as representative of TCs.

## Target projections of MCs and TCs

To visualize axon trajectories of MCs and TCs clearly, reporter proteins are expressed specifically only by olfactory bulb neurons that are neurogenic tagged, by combining neurogenic tagging and tetracycline-controlled transcription activation under the olfactory bulbs-specific promoter (Hirata et al., 2019; also see genotypes in the Figure 2 caption). Previously, TC axons were suggested to target only anterior region of the olfactory target areas (Haberly and Price, 1977; Scott, 1981; Igarashi et al., 2012). Our analysis revealed a surprising degree of convergence of TC axons; the axons only targeted to two small domains within the olfactory areas (Figure 2). One of the targets is the pars externa of the anterior olfactory nucleus, which uniquely receives topographic projections from the main olfactory bulb (Schoenfeld and Macrides, 1984). Its exclusive projections to the contralateral side suggest that the pars externa functions in bilateral integration of olfactory information (Haberly and Price, 1978b; Kikuta et al., 2010). The other TC target is the most anterolateral isolation of the CAP compartments (aiCAP) within the olfactory tubercle. Across the tubercle, dozens of CAP compartments are distributed in a patchy fashion, and their spatial locations vary between individual mice (Fallon et al., 1978; Meyer and Wahle, 1986). Among them, the aiCAP in the most anterolateral part of the tubercle consistently stands out as the largest and strongly expresses dopamine receptor 1. Notably, this small target receives TC projections from all glomeruli of the main olfactory bulb (Hirata et al., 2019).

MC axons exhibit a much more widespread distribution, projecting to all of the olfactory target areas (Figure 2). However, interestingly, the axons are specifically excluded from the aiCAP, making it a unique target exclusively innervated by TCs (Hirata et al., 2019). The pars externa appears to receive convergent inputs from both MCs and TCs, but the anatomical complexity of this subnucleus makes definitive conclusions challenging.

This observation provides compelling anatomical evidence for a dedicated TC-specific channel within the olfactory system. Although TC and MC projections were hypothesized to converge onto the same secondary target areas, the existence of the exclusive TC target offers exciting possibilities for TC-specific information processing within the olfactory system.

## Potential features represented by the TC Channel

What kind of features can be represented in the TC channel? As described already, TCs respond to a broad range of odorants at a low threshold (Schneider and Scott, 1983; Ezeh et al., 1993; Fukunaga et al., 2012; Igarashi et al., 2012). Combined with the fact that the aiCAP receives converging TC projections from all the olfactory bulb, this compact target could rapidly detect more-or-less indiscriminate odor stimuli. Because the aiCAP belongs to the dopamine reward system (Fallon et al., 1978; Haberly and Price, 1978a; Wesson and Wilson, 2011; Murata et al., 2015), the odor detection at aiCAP may directly influence value-based behavior in mice through this reward system.

The pars externa, another TC target, likely operates in partnership with MCs. Its unique topographic connections linking ipsilateral and contralateral olfactory bulbs suggest a potential role of this subnucleus in spatial function, such as the localization of odor sources (Kikuta et al., 2010).

## Future perspectives

The power of neurogenic tagging lies in its ability to manipulate the tagged neurons (Hirata et al., 2021). Thus, we are now in the stage to explore the actual meaning of olfactory parallel circuits. We have begun testing the olfactory behaviors of mice when MC or TC circuits are specifically activated or suppressed using chemogenetics. This method also paves the way for monitoring neuronal activities in various areas when neuronal activities of each olfactory channel is selectively modulated. Such approaches hold immense promise for unveiling the specific functions of MC and TC circuits within the olfactory system, ultimately leading to a deeper understanding of the logic behind olfactory information processing.

## Author contributions

TH: Writing – original draft, Writing – review & editing.

## Footnotes

^1^https://ssbd.riken.jp/neurogt/
